# Muslim women’s perspectives on the barriers to sexually transmitted infections testing and diagnosis in Saudi Arabia

**DOI:** 10.3389/fpubh.2023.1248695

**Published:** 2023-10-10

**Authors:** Noura Alomair, Samah Alageel, Nathan Davies, Julia V. Bailey

**Affiliations:** ^1^Community Health Sciences Department, College of Applied Medical Sciences, King Saud University, Riyadh, Saudi Arabia; ^2^Research Department of Primary Care and Population Health, Institute of Epidemiology and Health Care, University College London, London, United Kingdom

**Keywords:** religion, sexually transmitted infections, STI, HIV, testing, diagnosis, Muslim women

## Abstract

**Background:**

Muslim women are especially vulnerable to sexually transmitted infections (STI) and are at higher risk of under-detection. Evidence on the unique barriers to STI testing and diagnosis among Muslim communities is severely lacking. Understanding the complexity of accessing STI testing and diagnosis in Muslim communities is an area that requires further exploration. We aimed to explore the multilevel barriers to STI testing and diagnosis among Muslim women.

**Methods:**

We conducted qualitative semi-structured interviews informed by the ecological model of health. This study took place in Riyadh Saudi Arabia in 2019 with women aged over 18. Data were analysed using reflexive thematic analysis.

**Results:**

Twenty-eight women were interviewed from different ages, marital status, employment, and educational levels. Muslim women’s perceived barriers to STI testing and diagnosis included personal, socio-cultural, and service-level barriers. Lack of knowledge about STIs, denial, and underestimating the seriousness of infection and symptoms were among the many individual barriers to STIs testing and diagnosis. Religious views towards extramarital sex and negative views of people with STIs is a significant barrier to STI testing and diagnosis. Lack of patient confidentiality and providers’ judgement and mistreatment were also cited as barriers to seeking healthcare.

**Conclusion:**

Barriers to STI testing and diagnosis exist on many levels including personal, socio-cultural, religious, and healthcare services, and at policy level. Multilevel interventions are needed to reduce the stigma of STIs and facilitate access to sexual health services among Muslims. It is our recommendation that policy and research efforts are directed to enhance trust in the doctor-patient relationship through better clinical training as well as implementing stricter regulations to protect patients’ confidentiality in healthcare settings.

## 1. Introduction

Sexually transmitted infections (STIs) are one of the most common acute infections worldwide, and continue to be a major public health concern (1). In 2020, an estimated 37.7 million people are living with HIV worldwide, with 1.5 of HIV infections acquired in the year 2020 alone (2). Although most STIs are not life-threatening, they impose a tremendous economic and social cost worldwide (3). Left untreated, STIs can have a major impact on health including infertility, neonatal complications, and increased risk of cancer (4).

There are many barriers to STI testing and diagnosis. Some barriers are related to the structure of healthcare services, while other barriers are due to stigma, fear, and embarrassment (5). Denial of the existence of STIs is one of the most significant barriers to tackling the spread of infections in Muslim communities (6). STIs are viewed as a disease brought from non-Muslim countries in which sexual relations outside marriage are common and accepted (7). As such, there is a significant risk of under detection among religious communities as people do not view themselves as being at risk.

The Middle East and North Africa (MENA) region is predominantly made up of Islamic countries. Muslim women are especially vulnerable to under-detection of STIs (8). Many factors contribute to women’s vulnerability including marriage patterns (e.g., age gap between spouses, polygamy), and cultural expectations of women’s purity and innocence which means prohibition of extramarital sex is more strictly applied to women (7). Muslim women are less likely to accept HIV testing compared to Muslim men (8, 9). The MENA region can also be characterised by gender inequality and women with an STI are subjected to more judgement and discrimination than men (7, 10). This adds to the challenges women face accessing STI testing and treatment.

In Muslim countries like Saudi Arabia, STIs are one of the most under-recognised health issues (8). Saudi Arabia is characterised by low rates of HIV and other STIs (11), but STI rates are likely to be significantly underreported, with many cases undocumented. There is very limited data on STIs in Saudi Arabia as in many Islamic countries (8). It is presumed that rates of STIs are low due to the cultural and religious intolerance of extra-marital sex (12).

Research around STIs and HIV in the MENA region and other Islamic countries is mainly focused on knowledge, attitudes towards STIs, and the views towards people living with HIV. Understanding the complexity of accessing STI testing and diagnosis among Muslims is an area that requires further exploration. Due to the specific vulnerability of Muslim women, exploring barriers to STI testing and diagnosis among them is critical. Further research is needed to inform public health policies and interventions aimed at promoting the uptake of testing for STIs in religious communities and addressing the target population’s needs and concerns. Therefore, this study aimed to explore any personal, socio-cultural, and religious barriers, as well as service level barriers including healthcare providers and policy level barriers to STI testing and diagnosis among Muslims.

## 2. Methods

### 2.1. Study design

**Figure 1:**
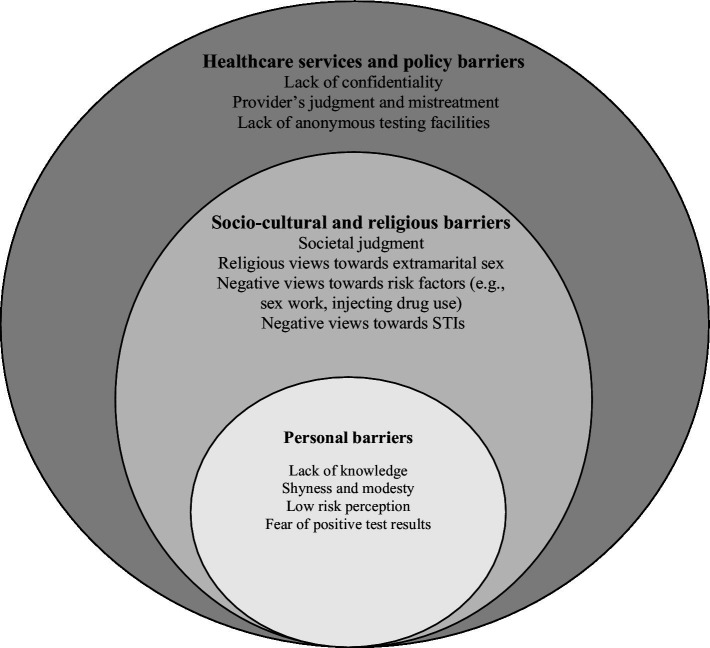
Barriers to STI testing and early diagnosis organised according to the conceptual framework.

We conducted a qualitative study to gain a deeper understanding of the barriers to accessing STI testing and diagnosis from Muslim women’s perspectives. This study was informed by a conceptual framework based on a modified version of the ecological model of health (13). The framework suggests that health is influenced by multiple factors and considers the interplay between personal, societal, and community, structural factors, as well as policies and regulations. The framework provided a roadmap for a comprehensive exploration of this sensitive topic (Figure 1). This study was approved by the UCL ethics committee (Reference no. 10157/001) and the hosting hospital in Riyadh, Saudi Arabia (Reference no. FWA00018774).

### 2.2. Recruitment and sampling

Recruitment took place in a public hospital in the women’s health outpatient clinic in Riyadh, Saudi Arabia, between January and June 2019. This hospital is one of the major public hospitals in Riyadh that provides primary, secondary, and tertiary healthcare services to the public free of charge.

Following a purposive sampling strategy, we recruited women from different age groups, marital statuses, educational levels, and employment. This heterogeneous sample allowed for the capturing of a wide variety of views and experiences. The recruitment process was done by the lead author (NA), a young Saudi female public health researcher. Potential participants were approached in the waiting areas of the hospital’s outpatients’ clinics where they were invited to take part in the study. Clinicians and clinic staff working in the hospital were able to provide support with the recruitment process, by asking patients in their clinics if they were interested to participate in the study, explaining the research aims, and handing out information sheets.

Before the start of each interview, participants signed the consent form and were given the chance to ask questions about the study. Participants were assured of the research team’s commitment to preserving their confidentiality and anonymity. We continued to recruit potential participants until no further themes were emerging and it was decided that thematic saturation was reached (14).

### 2.3. Data collection

One-to-one, face-to-face interviews were conducted, using a semi-structured interview guide developed by the research team. The interviews were conducted in Arabic, the participants’ native language, to allow participants to express their views with ease and confidence.

The topic guide was developed based on systematic reviews of the literature on Muslim women’s sexual and reproductive health (10, 15). Questions aimed to explore barriers and facilitators to STI testing and early diagnosis. The interview topic guide can be found in Additional file 1.

The interviews were conducted by the lead author (NA). The interviewer had no prior relationship with the study participants. All interviews were conducted in a private room in the hospital’s outpatient clinics. The interviews were audio-recorded with participants’ permission and ranged from 30 to 60 min. Before each interview, participants were asked to complete a survey to collect socio-demographic characteristics, such as age, marital status, and employment.

### 2.4. Data analysis

All interview recordings were transcribed verbatim by a professional transcription company. The lead author (NA) checked all transcripts against the original recordings. Data was uploaded to ATLAS.ti for data management and coding. The transcripts were kept in their original language as cultural nuances and expressions were lost in translation, which were considered important for the analysis, and subsequent interpretation.

An inductive approach to analysis was applied where codes were generated from the data using coding and refinement of themes. Transcripts were coded line-by-line, generating a set of codes based on close reading of the transcripts. A random sample of interviews was coded by another member of the research team (SA). Codes from each transcript were revised, compared, discussed, and amendments were made, where appropriate. Categories were derived from grouping codes to create the analytical framework and the themes were produced through discussions among all researchers. All codes and categories were in English, and all relevant quotes were translated from Arabic to English to facilitate the analysis and interpretation of the study findings.

Rigour was enhanced by keeping a reflexive diary, offering a clear account of procedures used, providing evidence from the data for all interpretations made, comprehensive analysis of the whole dataset, analysis of deviant cases and disconfirming data, comparing data between and within cases in the dataset and comparing findings with other studies (14).

## 3. Findings

Twenty-eight women, aged between 20 and 50 years, participated in the study. Sixteen were married and twelve were unmarried, the majority of women (*n* = 18) were college educated and employed, with five unemployed, and five college students. Table 1 provides an overview of women’s characteristics.

**Table 1:** Key characteristics of study participants.

	*N* (%)
**Marital status**	
Married	16 (57%)
Single	9 (32%)
Divorced	3 (11%)
**Age**	
20–25	7 (25%)
26–30	3 (11%)
31–35	8 (28%)
36–40	7 (25%)
41–50	3 (11%)
**Number of children**	
0	14 (50%)
1–2	2 (7%)
3–5	11 (39%)
6	1 (4%)

Barriers to STI testing and diagnosis existed on several levels of the conceptual framework (Figure 1), including personal factors which can be influenced by socio-cultural, religious, healthcare policy, and healthcare services factors. Table 2 summarises the main themes and subthemes according to the conceptual framework.

**Table 2:** Summary of main themes and subthemes.

	Domain			
Themes and subthemes	Personal	Socio-cultural	Religious	Healthcare services and policy
**Theme: Personal barriers to STI testing and diagnosis**				
***Subtheme***				
*Awareness and misconceptions*	✓	✓	✓	✓
*Perceived risk of STI*	✓	✓	✓	
*Fear of positive test result*	✓	✓	✓	✓
*Shyness and modesty*	✓	✓	✓	
**Theme: Socio-cultural and religious barriers to STIs testing and diagnosis**				
***Subtheme***				
*Societal judgement*	✓	✓	✓	✓
*Fears of exposure*	✓	✓	✓	✓
**Theme: Healthcare services and healthcare provider barriers**				
***Subtheme***				
*Lack of patient confidentiality*	✓	✓		✓
*Healthcare providers’ judgement*	✓	✓	✓	✓
**Theme: Facilitators to STI testing and diagnosis**				
*Facilitators to STI testing and diagnosis.*	✓	✓	✓	✓

### 3.1. Personal barriers to STI testing and diagnosis

#### 3.1.1. Awareness and misconceptions

Lack of knowledge about STIs, low-risk perception, denial, underestimating the seriousness of the infection, and long-term complications were among the many barriers to STIs testing and diagnosis. Participants lacked knowledge about different STIs, modes of transmission, and symptoms. Most women only knew of HIV/AIDS and used AIDS and STIs interchangeably, with a lack of knowledge about other STIs. This lack of awareness acts as a barrier to testing and seeking medical care for a possible STI.

*“I have never heard of any sexually transmitted infection other than AIDS”.* **(P1, Married, 34 years old)**

*“I know AIDS but can’t think of any other [STI] off the top of my head …”* **(P12, Married, 25 years old)**

*“Syphilis is transmitted through sexual contact, but AIDS I think can be transmitted through saliva and sneezing… What scares me is that I don’t know how to protect myself against it”* **(P19, Married, 38 years old)**

Women had significant misconceptions regarding STI modes of transmission, symptoms, and treatment. The misconception that STIs are caused by a lack of personal hygiene made it difficult for some women to get help as they were concerned with being perceived as unclean. STIs were also linked with extramarital sex, which also added to taboos and sensitivities around testing and diagnosis.

*"It's not necessarily from sexual intercourse, maybe you used something… For example, from toilets, it's not your fault. Using toilets that aren't sanitary or cleaned properly or maybe you used things that don't belong to you."* **(P16, Single, 36 years old)**

*"It's a sensitive subject. As I told you, maybe because of the idea that STIs are associated with either lack of hygiene or extramarital relations. So it’s a taboo because it’s linked to these two reasons.”* **(P23, Married, 28 years old)**

#### 3.1.2. Perceived risk of STI

Many women perceive themselves to be at no risk of contracting an STI, which can cause delays in testing and diagnosis. Almost all women in the sample believed that men were most likely to engage in extramarital sex. Muslim women having pre/extramarital sex was believed to be unheard of in their community. Since extramarital sex is forbidden in Islam, women believe that religion is protecting them from STIs which also contributes to the low-risk perception.

*“We have religious immunity against sexually transmitted infections because our religion forbids sexual relations before marriage so that protects us. I think even the cases of AIDS here are transmitted from blood, not sexual contact.”* **(P21, Married, 25 years old)**

*“It’s uncommon here because it’s transmitted through forbidden relations, and thank God, we still are God-fearing Muslims”* **(P7, Married, 44 years old)**

#### 3.1.3. Fear of positive test result

Women discussed fear of getting tested, and fear of a positive test result, as a barrier to STI diagnosis. It is worth noting that women consistently used gender-specific pronouns (he/him) when speaking about people with an STI.

*“Maybe it's fear, fear of reality. Fear of facing the fact that I have this. Or maybe he is underestimating the illness. Thinking that it is something simple or thinking that no there is nothing wrong with me. It will go away on its own. I feel mainly not wanting to face reality.”* **(P12, Married, 25 years old)**

*“I think he [the husband] would be scared, scared that someone would talk about him, so I think this would scare them and prevent them from seeking help from the start. There’s also denial, even with minor issues, there’s always denial, and that stops people from seeking treatment. So, imagine if it’s something as big as this. People will certainly be afraid of knowing.”* **(P26, Married, 22 years old)**

#### 3.1.4. Shyness and modesty

Although participants knew where to seek testing for STIs and HIV, shyness and modesty were among the main barriers to seeking medical help regarding anything sexual in nature. Women were not comfortable with physical and gynaecological examinations, sometimes accepting extreme pain rather than seeking medical care.

*“My mother, to this day, is still shy, she has seven kids but is still shy. She would rather suffer. She could have a simple problem, and I keep telling her to go see a doctor. And she refuses. She keeps giving excuses and then says: ‘Just leave me alone, it's bad enough I endured being exposed during childbirth just because I had to.’ We keep trying to convince her, but she won’t do it.”* **(P17, Single, 27 years old)**

*“I think shyness would prevent her from getting help. Because after all, it is a sensitive area, it’s not easy to get tested. So shyness would be the first barrier. What if it’s something bad … You also don’t want the doctor questioning your morality”* **(P19, Married, 38 years old)**

### 3.2. Socio-cultural and religious barriers to STIs testing and diagnosis

#### 3.2.1. Societal judgement

Almost all women in the research interviews believed that fear of stigma and societal judgment hindered STI testing and diagnosis. Social unacceptability of extramarital sex and extremely negative views towards people living with HIV are significant barriers to STI testing. Some women indicated that social influences play the most significant role in STI testing compared to any other factor.

*“I think it would be difficult to get help. It would be very difficult for him. Because he is scared of society. Because society has no mercy. Society will not be kind to him. So, he might secretly get checked, if, and only if, he could guarantee confidentiality. He then might get checked. But it would have to be permanently removed from his medical records.”* **(P14, Married, 38 years old)**

Women mentioned that socially constructed taboos around sexual health pose a significant barrier to healthcare seeking, particularly for women. Sex and sexuality are often referred to as “*ayb*” topics which translate to shameful, inappropriate, and dishonourable. A person is expected to feel “*ayb*” when violating religious and social rules, especially with regard to sexual health.

*“He fears stigma. It’s a disgrace, whether it was a minor infection or a serious one. It relates to such a sensitive area that’s supposed to be ayb and shameful. That’s why they stay quiet and do nothing.”* **(P23, Married, 29 years old)**

*“**P16:** You know what prevents her from seeking help? It’s the concept of ayb in our culture that’s actually scary. When normal people feel something is wrong, they seek help, but not here, she’s too afraid.*

***NA:** Why do you think she would be afraid?*

***P16:** Because of our ayb culture. So even if her issue was not sexually transmitted, she would still fear people interpreting it this way.”* **(P16, Single, 36 years old)**

#### 3.2.2. Fears of exposure

Fear of being exposed through breaches in patient confidentiality, or being seen getting tested, was believed to be worse than having the infection and suffering the consequences. Avoiding societal judgement (making it easier for people to seek help) is believed to be impossible, as “*we live in an extremely connected society*”. Living independently of other people and being unconcerned with society’s opinion was considered abnormal.

*“I don’t think it would be easy to get checked. Because we have other problems. We are not like people from Western countries. Where everyone lives on their own, in their own home … We live a very social life. I’m with people from the moment I wake up to the moment I sleep. The only time I am on my own is when I am sleeping. So, we don't have someone that is anti-social. It is not an option. You have to have social interaction every moment of your day.”* **(P11, Divorced, 33 years old)**

*“He won’t go [get tested] because he’s afraid he’ll be exposed. To his wife, his family, this fear is worse than anything else including living with the disease.”* **(P5, Married, 43 years old)**

To avoid being recognised by someone they know and face the consequences of being exposed as having an STI, a participant suggested seeking healthcare privately and using false identification.

*“They should go to a private lab and either not give their ID or use a fake one. Because even if he was an educated person aware of the consequences of not seeking treatment, his fear of exposure and its consequences might outweigh his desire to be treated.”* **(P23, Married, 29 years old)**

### 3.3. Healthcare services and healthcare provider barriers

#### 3.3.1. Lack of patient confidentiality

A crucial barrier to STI testing is the lack of patient confidentiality. Many women highlighted that some healthcare providers do not respect doctor-patient confidentiality. The issue of compromised confidentiality was often because of healthcare providers discussing their work life in social settings.

*“Some people don’t respect what you call confidentiality. Even in healthcare, I always hear, for example, a doctor said: ‘Oh, I saw this patient [her name] today.’ I mean a patient you know; I’ve heard many stories. One of my friends was following up with a doctor, something not related to sexual health. And she knows the doctor’s daughter. So, the doctor told his daughter: ‘Your friend came to me today, and she has this and this and that.’ And I mean he is a doctor. He should take confidentiality more seriously. And it wasn't something sexual, it wasn't something Ayb, but still, he shouldn’t share.”* **(P25, Divorced, 32 years old)**

*“They are all concerned that someone would talk about them to someone. That would scare them and prevent them from seeking help. Even if they go to the hospital, they will call names out loud in the waiting area and everyone would know their name”* **(P26, Married, 22 years old)**

Although lack of confidentiality was viewed negatively, it was believed to be acceptable to breach patient confidentiality if the woman is wanting to know, particularly in the case of engagement or marriage.

*“Sadly, we have this issue with having an extremely social life. Everyone knows everyone. So, for example, my friend would come and tell me that I saw this person, getting treatment or tested for something. And I personally believe if that person is marrying a person that I know, I will let her know, because it is her right to know.”* **(P11, Divorced, 33 years old)**

Concerns over doctors sharing the diagnosis with spouses were raised by some women. Men would rather keep having sex with their wives and transmit an infection than tell them that they might have an STI.

*“Some people even keep this from their wives to the extent that they might even keep having sex and transmit the infection to her rather than tell her that he has been diagnosed with something … It's only a disease that, with time, will find a cure. But no, he will think this will compromise his manhood. He’ll think it is a scandal and no one should know about it.”* **(P23, Married, 23 years old)**

#### 3.3.2. Healthcare providers’ judgement

Healthcare providers’ judgement and mistreatment were cited as a barrier to STI and HIV healthcare seeking. Due to the socio-cultural unacceptability of STI risk factors, individuals seeking testing can be subjected to poor treatment or discrimination by healthcare providers.

*“We need healthcare providers to not look at patients with contempt and disdain or judge them to be a bad person. We need to have a safe space for patients. We also need to educate them about confidentiality.”* **(P19, Married, 38 years old)**

*“I noticed that doctors can be very judgemental sometimes. Of course, not all but some, and this judgement would make me closed off. I wouldn’t be honest.”* **(P26, Married, 22 years old)**

*“We fear judgement when seeking help for these things [STIs]. Because we believe these things are a punishment from God for committing forbidden acts”* **(P8, Single, 22 years old)**

#### 3.3.3. Facilitators to STI testing and diagnosis

Many participants suggested ways of improving STI testing by using codes instead of people’s names and identity documents when seeking medical care. They also suggested that test results should be removed from the patient’s permanent medical records, as it is possible for anyone working in the institution to access the patient’s medical records.

*“The problem in our community, family names are well-known, so anyone in the hospital might recognise the family name. I think maybe if we used numbers or codes instead of people’s names it would encourage more people to get tested and seek medical help.”* **(P8, Single, 22 years old)**

Women believed that it is preferable to go to a foreign doctor to protect patient privacy, explaining that it would be less likely for a non-Saudi doctor to recognize the family name, and therefore risk being exposed by someone they know.

*“They [healthcare providers] need to know that it is not okay to share patients’ information. We live in an interrelated community. People know each other. So, I feel that to avoid being exposed, people will seek non-Saudi doctors. Saudi doctors would know your family name, they might know someone that knows someone, and they could talk. You can’t even go in public without seeing someone you know. So, they could share the information without intentionally meaning to share it.”* **(P19, Married, 38 years old)**

To make it easier for people to access STI testing and diagnosis, some women suggested that people should seek treatment outside the country to avoid societal judgement and risk exposure.

*“Because we are so fearful of society’s judgement, if the person is educated and is concerned about his health, it might actually be easier for them to seek help outside the country. Whether it was testing or treatment. He might need to leave the whole society to seek help.”* **(P23, Married, 28 years old)**

## 4. Discussion

Barriers to STI and HIV testing and diagnosis exist on many levels including personal, socio-cultural, religious, healthcare providers, and policy level barriers. Personal factors include lack of knowledge about STIs, denial, low-risk perception, fear of a positive result, and fear of being exposed due to breaches in confidentiality. Consistent with our findings, fear of a positive test result and fear of the disease were reported among the most significant barriers to testing and diagnosis (5). The perception that HIV is a deadly infection rather than a chronic manageable illness is a major part of that fear. A study from the United States reported that fear was the most cited reason for delayed testing, as participants were ‘afraid of the answer’ (16). People who engaged in high-risk behaviours (e.g., injecting drug use) were more likely to be afraid of getting tested (16).

Our research revealed that Muslims believe that they have “religious immunity” against STIs. This was consistent with previous literature where the low perception of risk was influenced by strong religious beliefs (6, 15, 17). Many Muslims believe that conforming with religious values offers the best protection from STIs (6, 8, 15). HIV testing among Muslims is consistently lower compared to other religions, which is believed to contribute to the spread of STIs among Muslim communities (8, 18, 19).

Low perception of risk can lead to significant delays in STI diagnosis, particularly for married women who have never engaged in premarital sex. For many Muslim women, their husbands are their first and only lifetime sexual partners, so they assume that they are at no risk of contracting an STI (8, 20–22). Marriage was reported as the main occasion for the transmission of HIV among Muslim women (8, 21–24). The majority of married women living with HIV were diagnosed either during their first pregnancy as a result of routine antenatal testing, when their children became seriously ill and tested positive for HIV, or when their husbands disclosed that they are HIV positive (8).

Women in this research explained that people in Saudi Arabia are afraid of seeking medical care for a possible STI out of fear of being exposed either by healthcare providers or by someone in the healthcare facility. Worries about being exposed directly through breaches in patient confidentiality, or indirectly by being seen getting tested, is a significant obstacle to STI diagnosis and treatment (5, 25–27). Fear of breaches in patient confidentiality is one of the most common barriers to HIV testing among sexually active youth (26–28). Fear of disclosure of an STI diagnosis to family members is not unjustified, and concerns over the lack of patient confidentiality in medical settings has been previously reported in the MENA region (29–31).

Healthcare providers’ prejudice, judgement, and mistreatment of people with an STI can prevent at-risk individuals from getting tested and seeking treatment. Healthcare providers’ judgement and discrimination against people living with HIV have been consistently reported in the literature as a barrier to HIV testing (27, 32). Our research shows that healthcare providers religious views towards STI risk factors (i.e., extramarital sex, drug use) influenced their treatment of individuals with an STI.

Our research revealed that stigma and shame associated with HIV and STIs acted as a barrier to testing and diagnosis. Stigma is a major obstacle to combating HIV/AIDS globally (26, 33–35). The majority of infections are among sex workers, injecting drug users or men who have sex with men, all of which are forbidden by religion and illegal in most countries in the region (8, 26). Those groups are forced to conceal their lifestyle out of fear of being physically harmed, punished, or ostracized from society (8, 34). This stigma makes it hard, and frightening, for those individuals to seek counselling and testing, or to disclose their diagnosis to anyone in their community (26, 34).

### 4.1. Strength and limitations

This is the first qualitative study, to the authors knowledge, to explore barriers to STI testing and diagnosis in the MENA region. Our findings are transferable to Muslim communities around the world, as many Muslims share similar cultural values and traditions (36–39). Interviewing women from different age groups and marital statuses allowed for comparisons to be made across different experiences, offering an opportunity to explore the narratives of unmarried women. In sexual health research, it is uncommon to interview or survey unmarried Muslim women in Islamic countries.

Qualitative interviews were conducted in Arabic, the participants’ native language. Some cultural references are not easily translated, and some of the meanings might have been lost in translation. Yet, the translation process can analytically productive and a critical challenge that can add to interpretations (40, 41).

Face-to-face interviews discussing sensitive topics (i.e., sexual health) have the potential to introduce social desirability bias. Guaranteeing the privacy of the participants and ensuring confidentiality throughout the interviews encourages participants to provide their honest accounts.

### 4.2. Implications for policy and practice

One of the reasons for delayed STI and HIV testing is a lack of knowledge and understanding of STIs. It is therefore essential that healthcare providers carefully assess their patients’ understanding of common STIs and the presentation of symptoms, offering advice and tests accordingly.

One of the most important barriers to early STIs testing and diagnosis is concern over breaches in patient confidentiality and fear of exposure. It is crucial to enforce stricter codes of practice against breaches in patient confidentiality by all staff in a healthcare setting. It is our recommendation that policy, research, and training programs are developed to promote the public’s trust in doctor-patient professional relationships and confidentiality in healthcare settings.

Although there are currently sexual health clinics offering anonymous testing in Saudi Arabia, those clinics are private and tests are expensive, making them inaccessible to everyone (42). It would therefore be useful to provide anonymous free testing clinics to encourage individuals to get tested without fear of exposure.

Extensive public health efforts need to be directed toward reducing the stigma and discrimination against people living with HIV/AIDS among Muslim communities. The extreme stigma and discrimination towards high prevalence groups (e.g., sex workers, injecting drug users), act as a significant barrier to seeking testing and treatment. It is imperative to develop programs directed toward the general population to tackle stigma, shame, and judgement. Professional training programs and national policies and regulations should be developed to improve access to sexual health services for all members of society regardless of personal beliefs or behaviours. This needs to be explicitly emphasised in healthcare settings and among service providers.

### 4.3. Future research

Better quality studies on the barriers to STI testing and diagnosis, particularly in the MENA region, are needed. However, conducting high-quality research can be difficult in some countries due to restrictions on data sharing and reporting (43, 44). Improved reporting and better surveillance are essential for accurate STI and HIV prevalence estimates to inform public health policies and prevention measures.

## 5. Conclusion

Barriers to STI testing and diagnosis exist on many levels including personal, socio-cultural, religious, healthcare settings and policy level barriers. The results suggested that lack of knowledge, fear, confidentiality breaches, and stigma associated with STIs act as barriers to early testing and treatment. There is a need for multilevel interventions to facilitate access to sexual health services and reduce the stigma attached to STIs and HIV in Muslim communities. It is our recommendation that policy and research efforts are directed to promote the public’s trust in the doctor-patient relationship and implement stricter regulations to protect patients’ confidentiality in healthcare settings.

## Data availability statement

The datasets presented in this article are not readily available because of confidentiality concerns. Requests to access the datasets should be directed to UCL ethics committee: ethics@ucl.ac.uk.

## Ethics statement

The studies involving humans were approved by the UCL ethics committee (Reference no. 10157/001) and the hosting hospital in Riyadh, Saudi Arabia (Reference no. FWA00018774). The studies were conducted in accordance with the local legislation and institutional requirements. The participants provided their written informed consent to participate in this study.

## Author contributions

NA, SA, ND, and JB: conceptualization and writing – review and editing. NA: data curation. NA and SA: formal analysis and writing – original draft. NA, ND, and SA: methodology. All authors contributed to the article and approved the submitted version.
