# Supplementary material for: Muslim women’s perspectives on the barriers to sexually transmitted infections testing and diagnosis in Saudi Arabia
Source: Front Public Health. 2023 Oct 10;11:1248695. doi: 10.3389/fpubh.2023.1248695 (PMC10595004; doi:10.3389/fpubh.2023.1248695)
Supplement: Supplementary file 1 [file Table_1.DOC]

**Additional file 1: Topic guide**

| **Conceptual framework level** | **Questions guide** |
| --- | --- |
| **Personal barriers (knowledge)** | Awareness of different sexually transmitted infections (STIs)  Awareness of signs and symptoms  Awareness of prevention measures  Awareness of when to get tested  Awareness of testing facilities/where to get tested |
| **Personal barriers (beliefs and perceptions)** | Perceptions of personal risk  Perceived seriousness of infection  Would you get tested?  Who should/should not get tested?  Would it be easy/difficult?  Who is at risk of acquiring an STI |
| **Society, culture, and religion** | Are STIs a concern in Saudi Arabia?  Religious beliefs and risk of STIs  Ease/difficulty of getting tested for a possible STI  Would it be easier to get tested in your society?  Why would it be easy/difficult?  What would make it easier to get tested for an STI? |
| **Healthcare services and Healthcare providers** | Healthcare facility (Public or private)  Healthcare facility (structure)  Sense of ease and safety with healthcare providers  Sense of trust disclosing risk behaviours to healthcare providers  Anonymity and confidentiality  Providers characteristics (age, gender, nationality)  Trust in healthcare providers |
